# Supplementary material for: Synthesis and Antimicrobial Activity Evaluation of Homodrimane Sesquiterpenoids with a Benzimidazole Unit
Source: Molecules. 2023 Jan 17;28(3):933. doi: 10.3390/molecules28030933 (PMC9921711; doi:10.3390/molecules28030933)

SYNTHESIS AND ANTIMICROBIAL ACTIVITY EVALUATION OF HOMODRIMANE SESQUITERPENOIDS WITH BENZIMIDAZOLE UNIT

Lidia Lungu[1], Svetlana Blaja[1], Caleria Cucicova[1], Alexandru Ciocarlan[1], Alic Barba[1], Veaceslav Kulcițki[1], Sergiu Shova[2], Nicoleta Vornicu[3], Elisabeta-Irina Geana[4], Ionel I. Mangalagiu[5], and Aculina Aricu[1*]

1 Chemistry of Natural and Biologically Active Compounds Laboratory, Institute of Chemistry, 3 Academiei Str., MD-2028 Chisinau, Moldova; lidilungu@yahoo.com (L.L.); svetlana-blaja@mail.ru (S.B.); cucicovac@yahoo.com (C.C.); algciocarlan@yahoo.com (A.C.); alicbarba@gmail.com (A.B.); kulcitki@yahoo.com (V.K.); aculina.aricu@gmail.com (A.A.)

2 *"P. Poni"* Institute of Macromolecular Chemistry, Aleea Grigore Ghica Voda 41-A, 700487 Iasi, Romania; shova@icmpp.ro (S.S.)

3 Metropolitan Center of Research T.A.B.O.R., 9 Closca Str., RO-700066 Iasi, Romania; cmctaboriasi@yahoo.com (N.V.)

4 Department of Research and Development, National Research and Development Institute for Cryogenics and Isotopic Technologies—ICSI Rm. Valcea, 4th Uzinei Str., PO Raureni Box 7, 240050 Rm. Valcea, Romania; irina.geana@icsi.ro (E.-I.G.)

5 Faculty of Chemistry, "*Alexandru Ioan Cuza*" University of Iasi, 11 Carol Bd., Iasi RO-700506, Romania; ionelm@uaic.ro (I.I.M.)

* Correspondence: aculina.aricu@gmail.com (A.A.)

SUPPLEMENTARY INFORMATION

Table S1. Bond distances (Å) and angles (Å) for 20.

C4-C14	1.528(6)	C5-C6-C7	110.2(3)
C4-C15	1.531(6)	C8-C7-C6	112.5(3)
C5-C6	1.525(4)	O1-C8-C7	108.5(3)
C5-C10	1.559(4)	O1-C8-C9	101.2(2)
C6-C7	1.534(5)	O1-C8-C11	107.7(3)
C7-C8	1.515(5)	C7-C8-C9	109.9(3)
C8-C9	1.562(4)	C7-C8-C11	113.8(3)
C8-C11	1.534(6)	C11-C8-C9	114.8(3)
C9-C10	1.570(5)	C8-C9-C10	114.5(2)
C9-C16	1.544(5)	C16-C9-C8	112.3(3)
C10-C24	1.538(5)	C16-C9-C10	112.4(3)
C12-C13	1.479(7)	C1-C10-C5	107.0(3)
C16-C17	1.513(5)	C1-C10-C9	108.2(2)
C20-C19	1.39	C5-C10-C9	107.4(2)
C20-C21	1.39	C24-C10-C1	107.9(3)
C19-C18	1.39	C24-C10-C5	114.3(3)
C18-C23	1.39	C24-C10-C9	111.8(3)
C23-C22	1.39	O1-C12-C13	110.8(4)
C22-C21	1.39	O2-C12-O1	125.0(4)
C12-O1-C8	122.5(3)	O2-C12-C13	124.2(4)
C17-N1-C18	125.4(8)	C17-C16-C9	116.4(3)

C2-C1-C10	113.4(3)	O3-C17-N1	122.5(3)
C3-C2-C1	111.6(3)	O3-C17-C16	122.9(3)
C2-C3-C4	114.2(3)	C19-C18-N1	114.3(10)
C3-C4-C5	107.2(3)	C23-C18-N1	125.7(10)
C14-C4-C3	110.7(3)	N1-C17-C16	114.4(3)
C14-C4-C5	114.6(3)	C20-C19-N2	112(2)
C14-C4-C15	107.8(3)	C18-C19-N2	127(2)
C15-C4-C3	107.4(3)	C23-C18-C19	120
C15-C4-C5	109.0(3)	C18-C23-C22	120
C6-C5-C4	113.7(2)	C23-C22-C21	120
C6-C5-C10	110.8(3)	C22-C21-C20	120
C10-C5-C4	117.3(3)	C18-C19-C20	120
		C19-C20-C21	120

Table S2. Selected crystallographic data refinement parameters for 20.

Formula	$C_{24}H_{36}N_2O_3$
Fw	400.55
Space group	$P2_1$
a [Å]	8.2121(3)
b [Å]	9.6498(3)
c [Å]	14.9502(5)
α [°]	90
β [°]	105.745(4)
γ [°]	90
V [Å^3]	1140.28(7)
Z	2
r_{calcd} [g cm^{-3}]	1.167
Crystal size [mm]	0.35×0.15×0.10
T [K]	294
μ [mm^{-1}]	0.076
2Θ range [°]	5.082 to 52.744
Ref. collected	14240
Indep.refl. R_{int}	4447, 0.0248
Data/restr./param.	4447/2/253
R_1	0.0578
wR_2	0.1573
GOF	1.036
CCDC	2225381

^{1}H NMR spectrum of compound 4

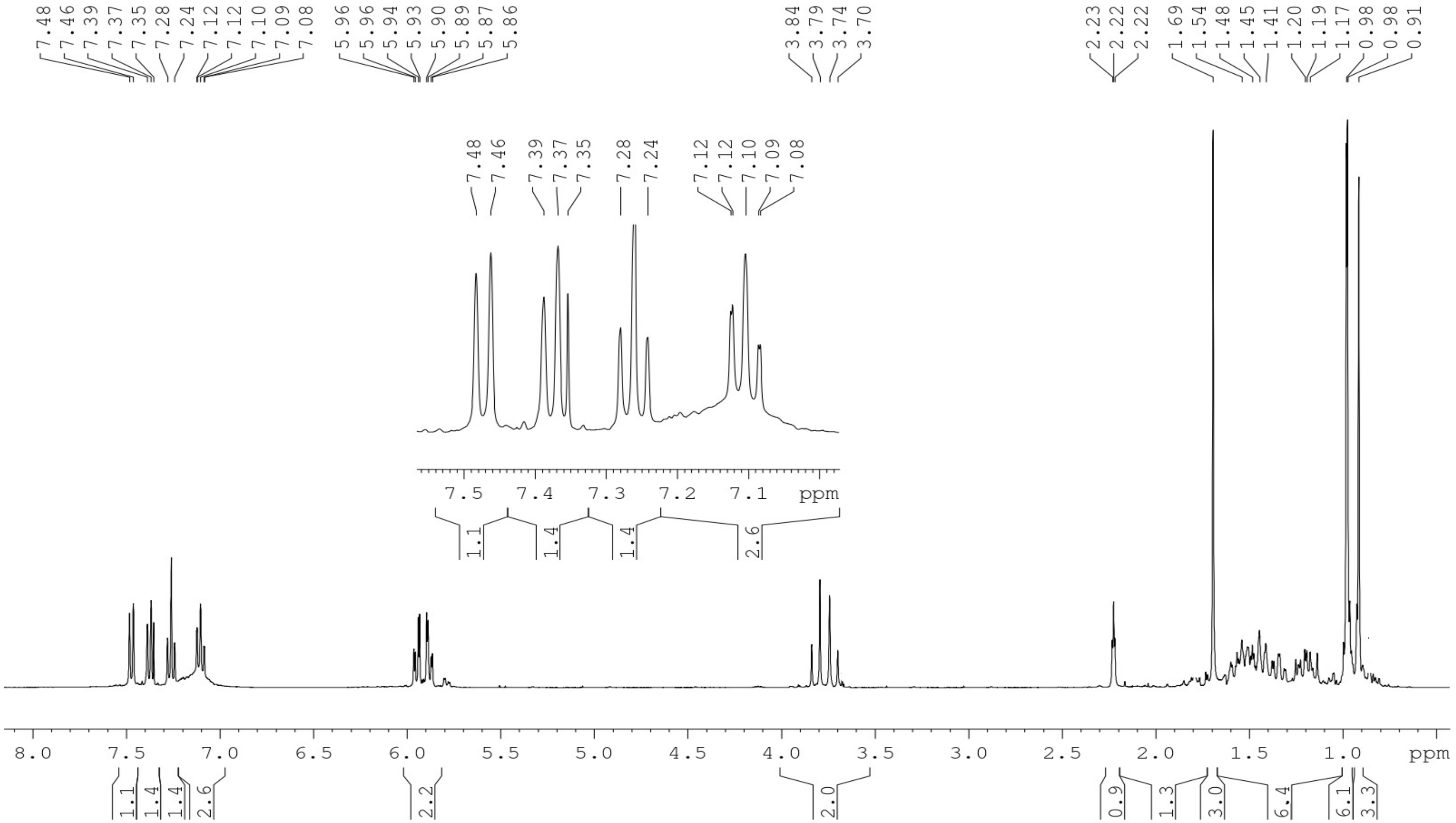

^{13}C NMR spectrum of compound 4

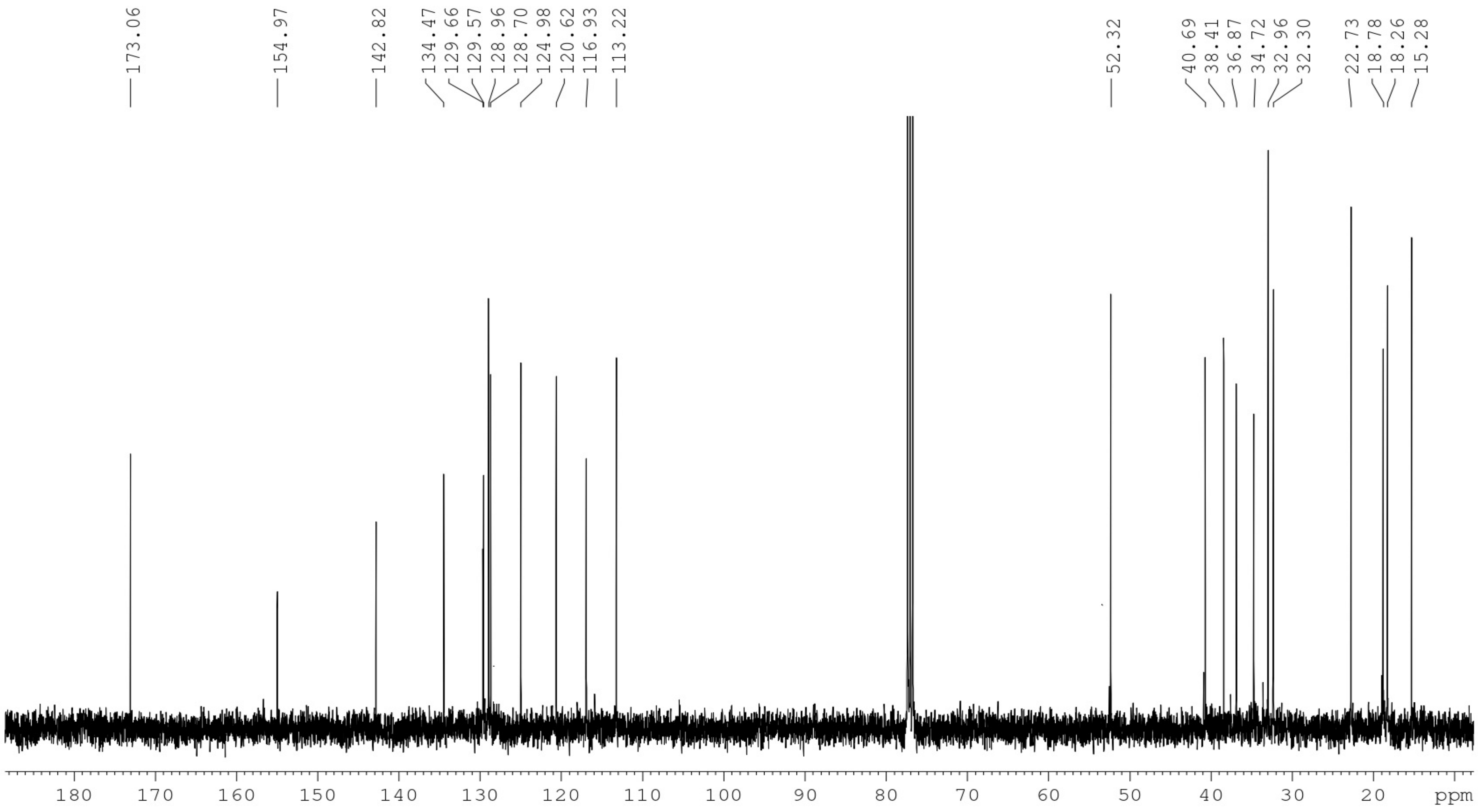

^{1}H NMR spectrum of compound 7

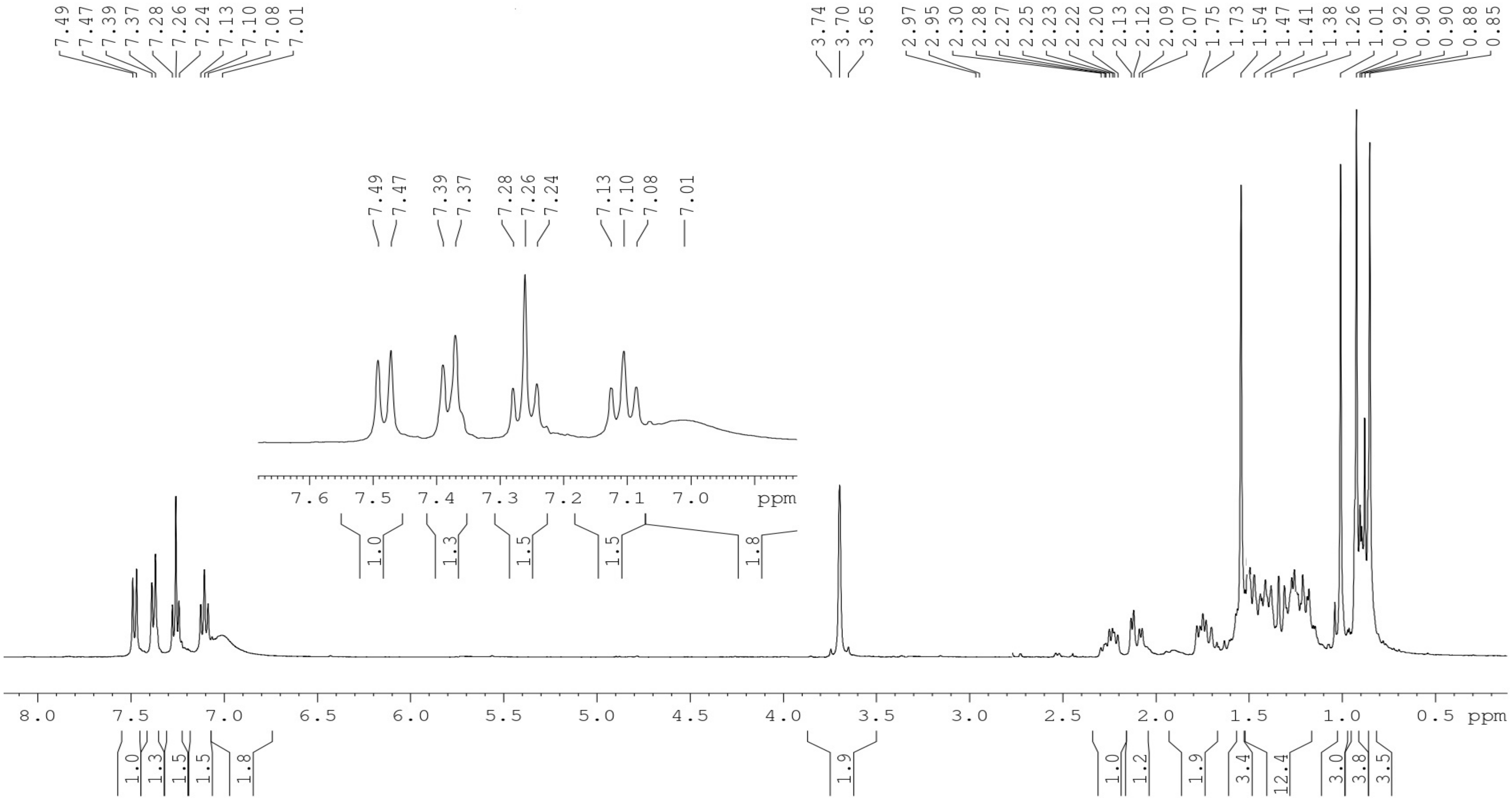

^{13}C NMR spectrum of compound 7

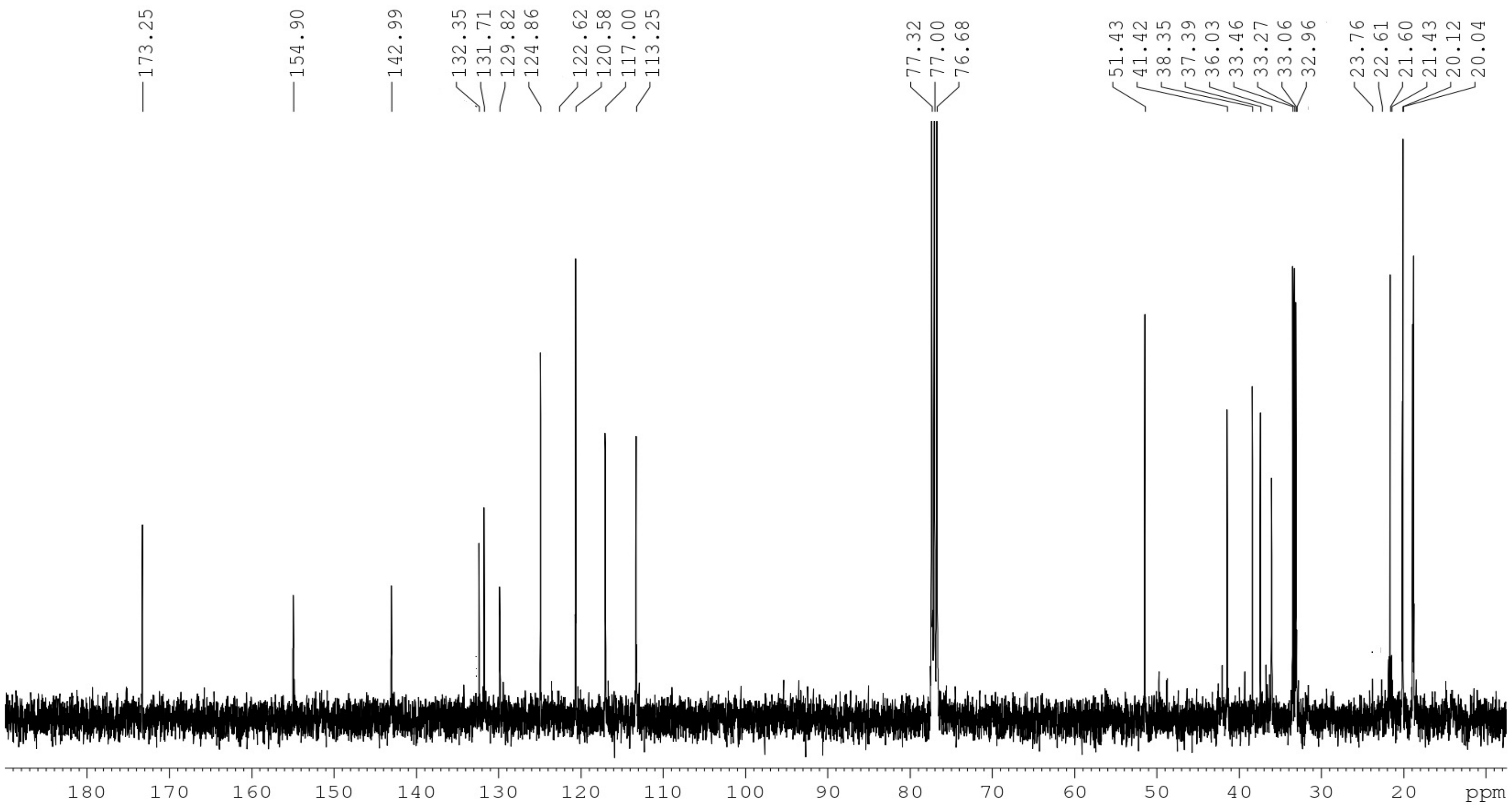

^{1}H NMR spectrum of compound 10

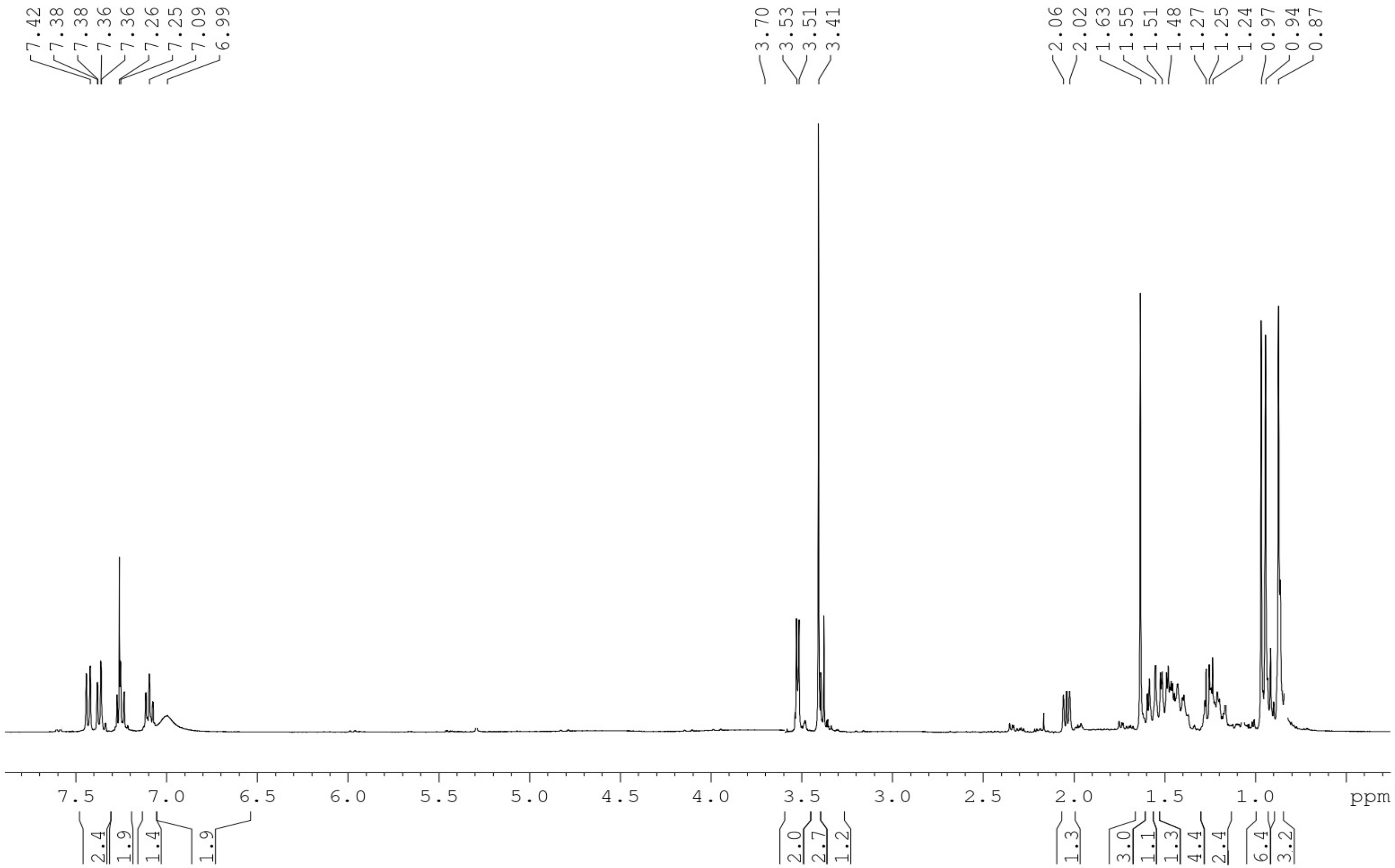

^{13}C NMR spectrum of compound 10

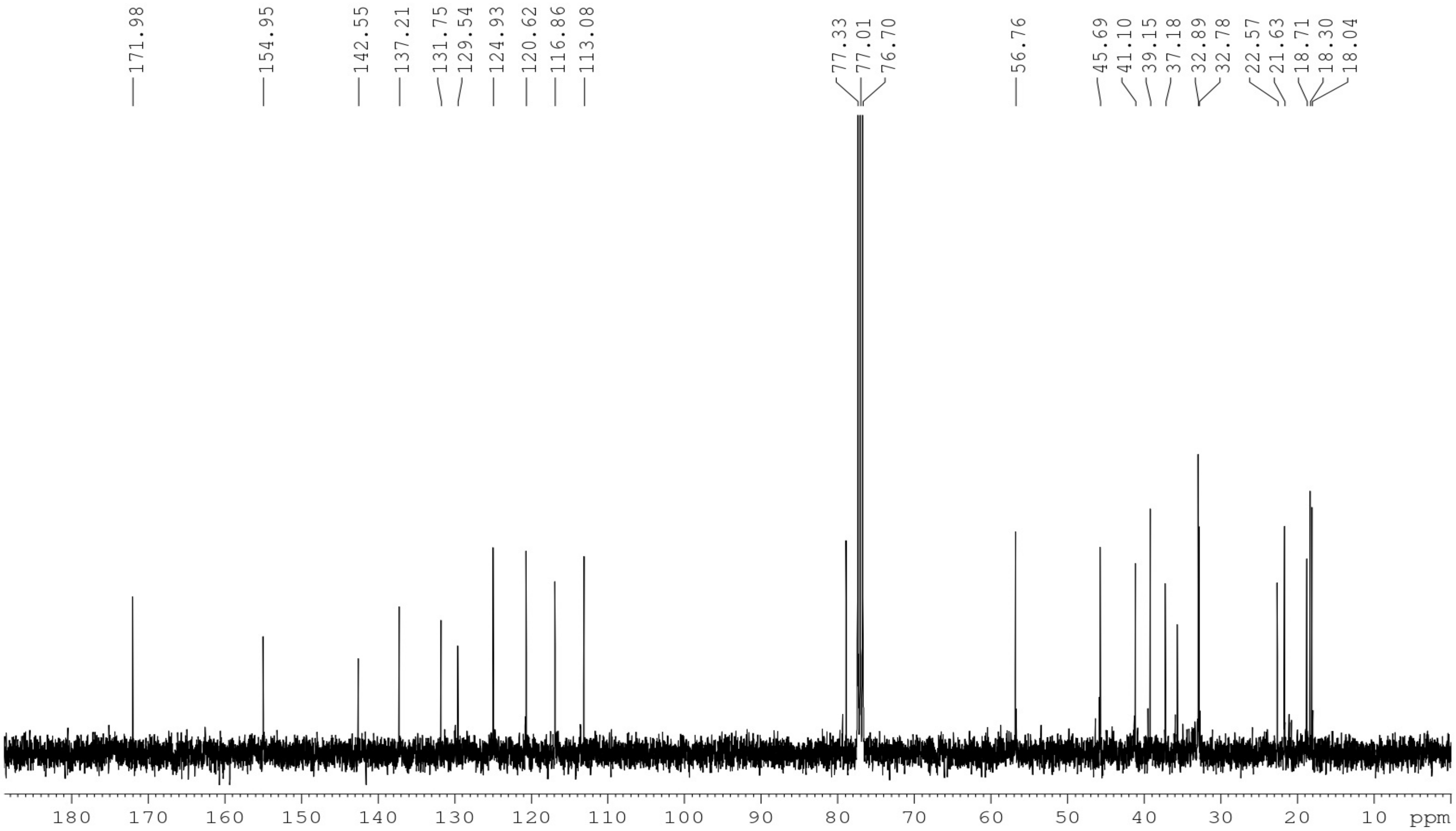

^{1}H NMR spectrum of compound 14

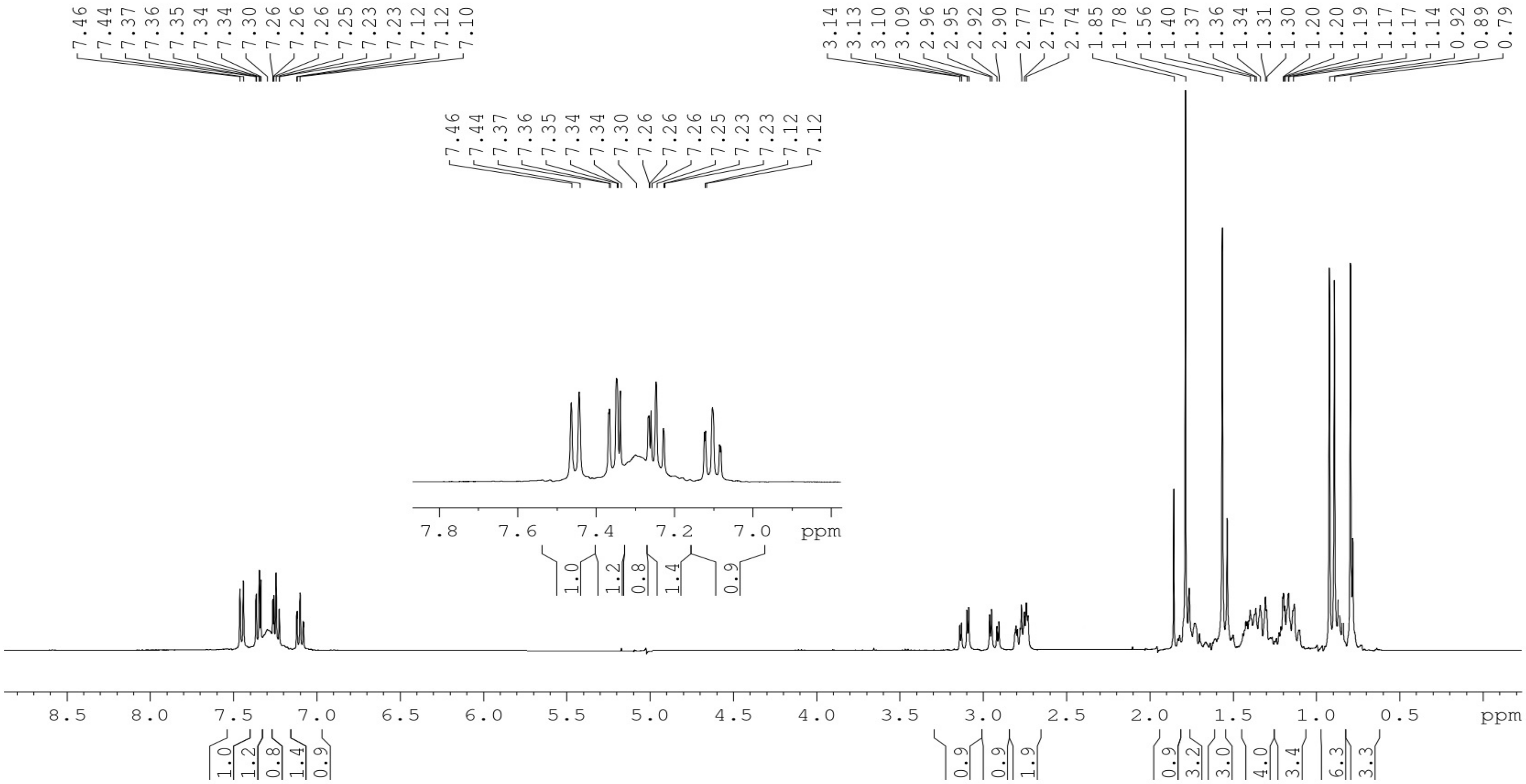

^{13}C NMR spectrum of compound 14

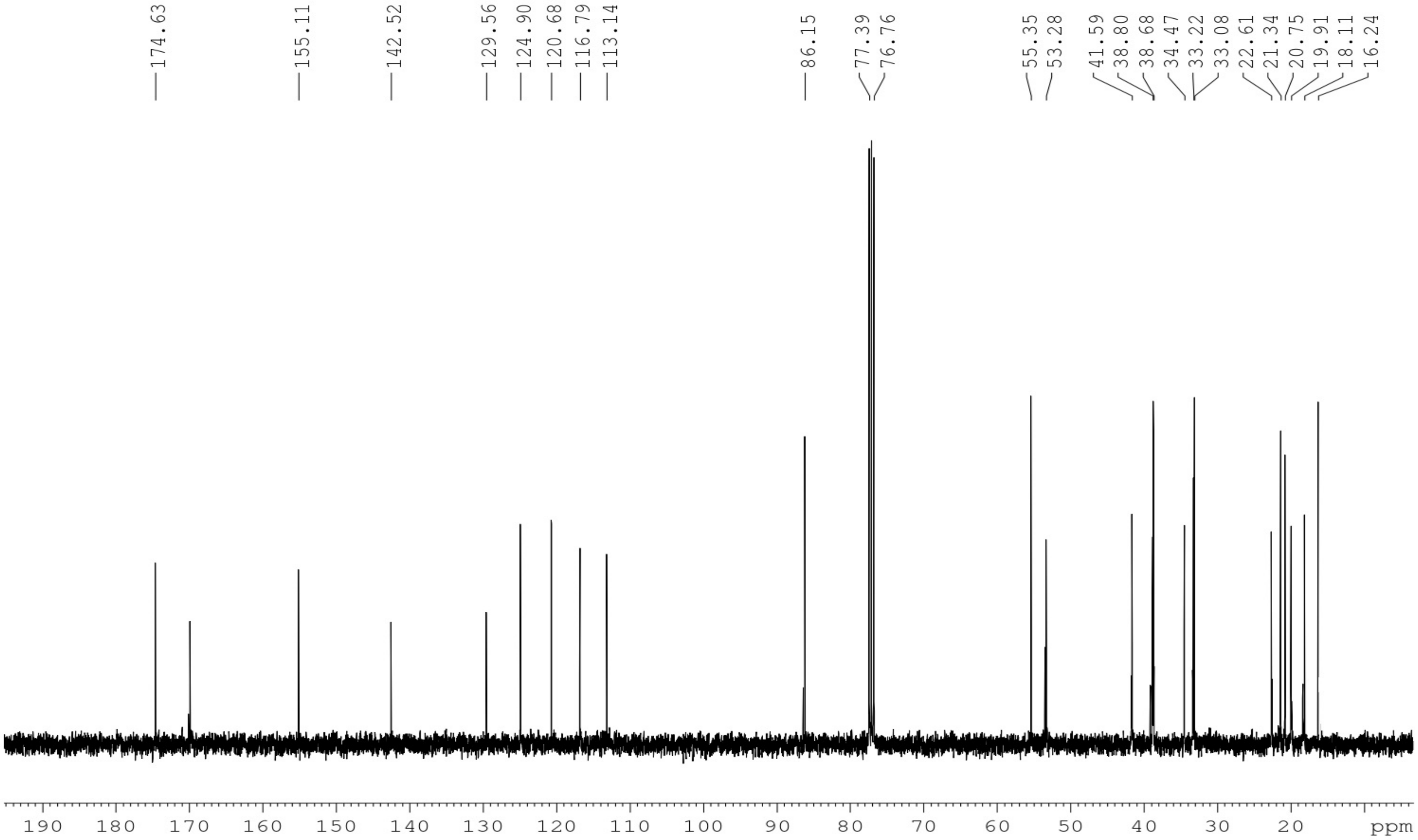

¹H NMR spectrum of compound 15

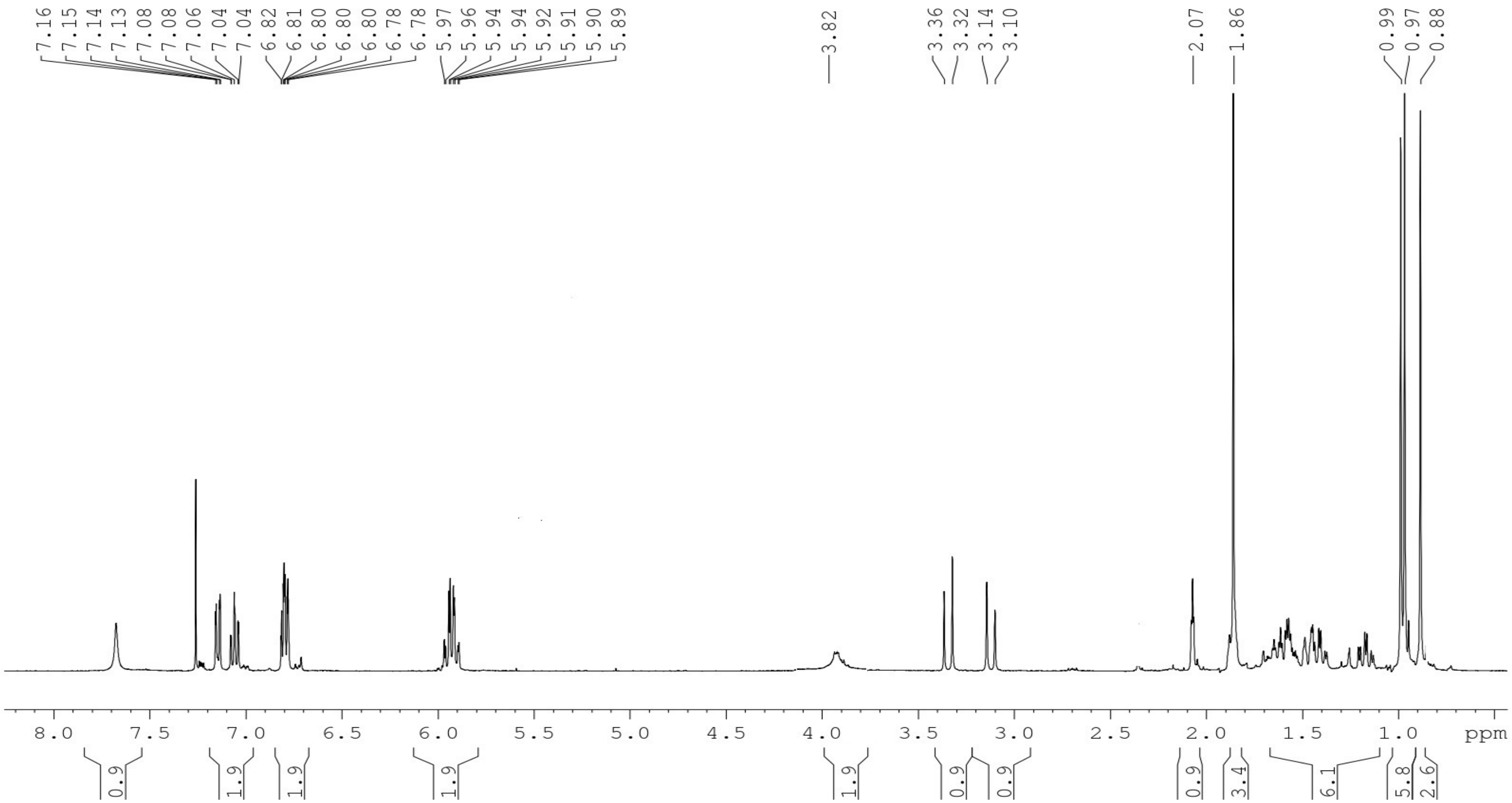

^{13}C NMR spectrum of compound 15

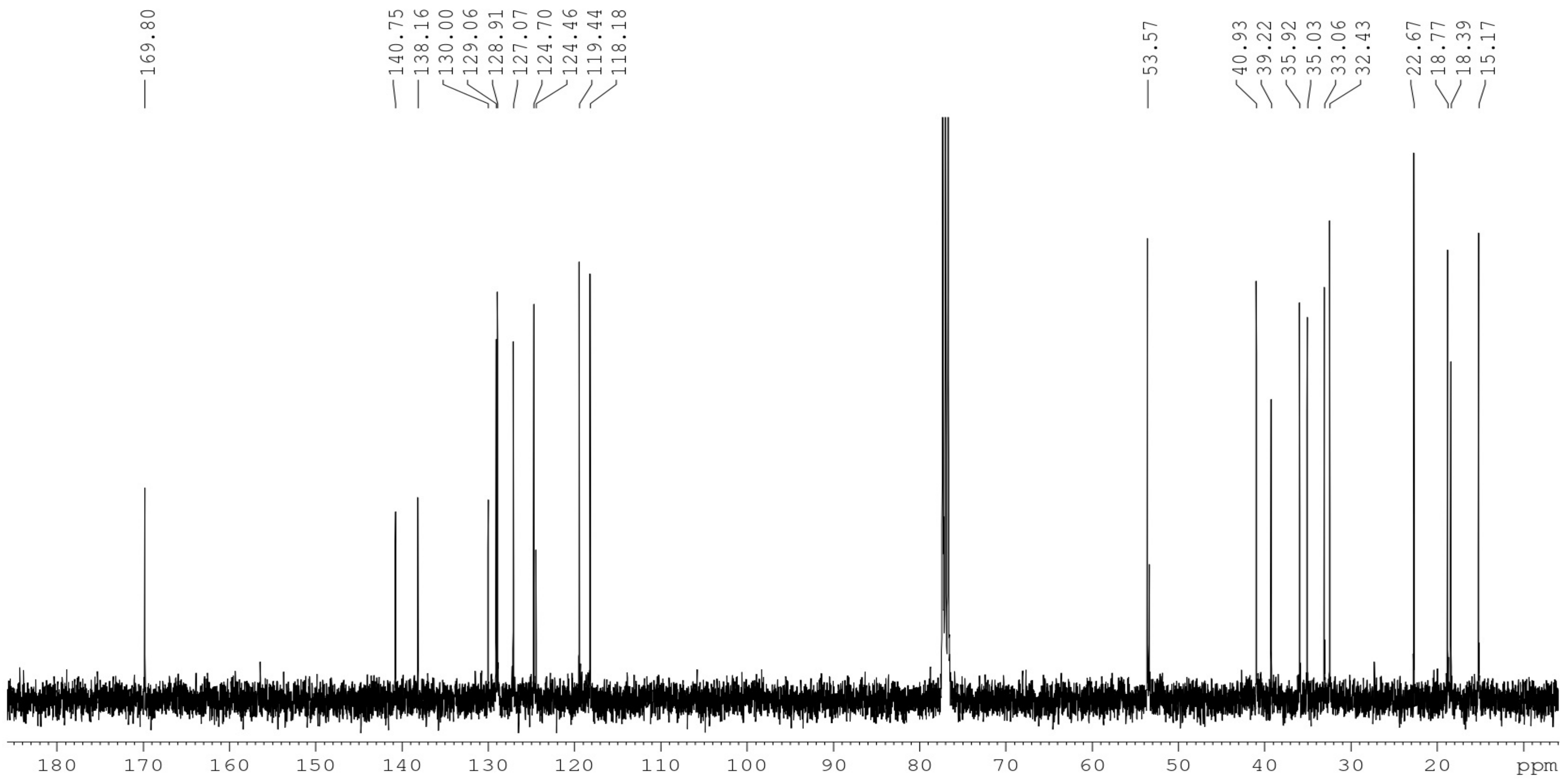

^{1}H NMR spectrum of compound 16

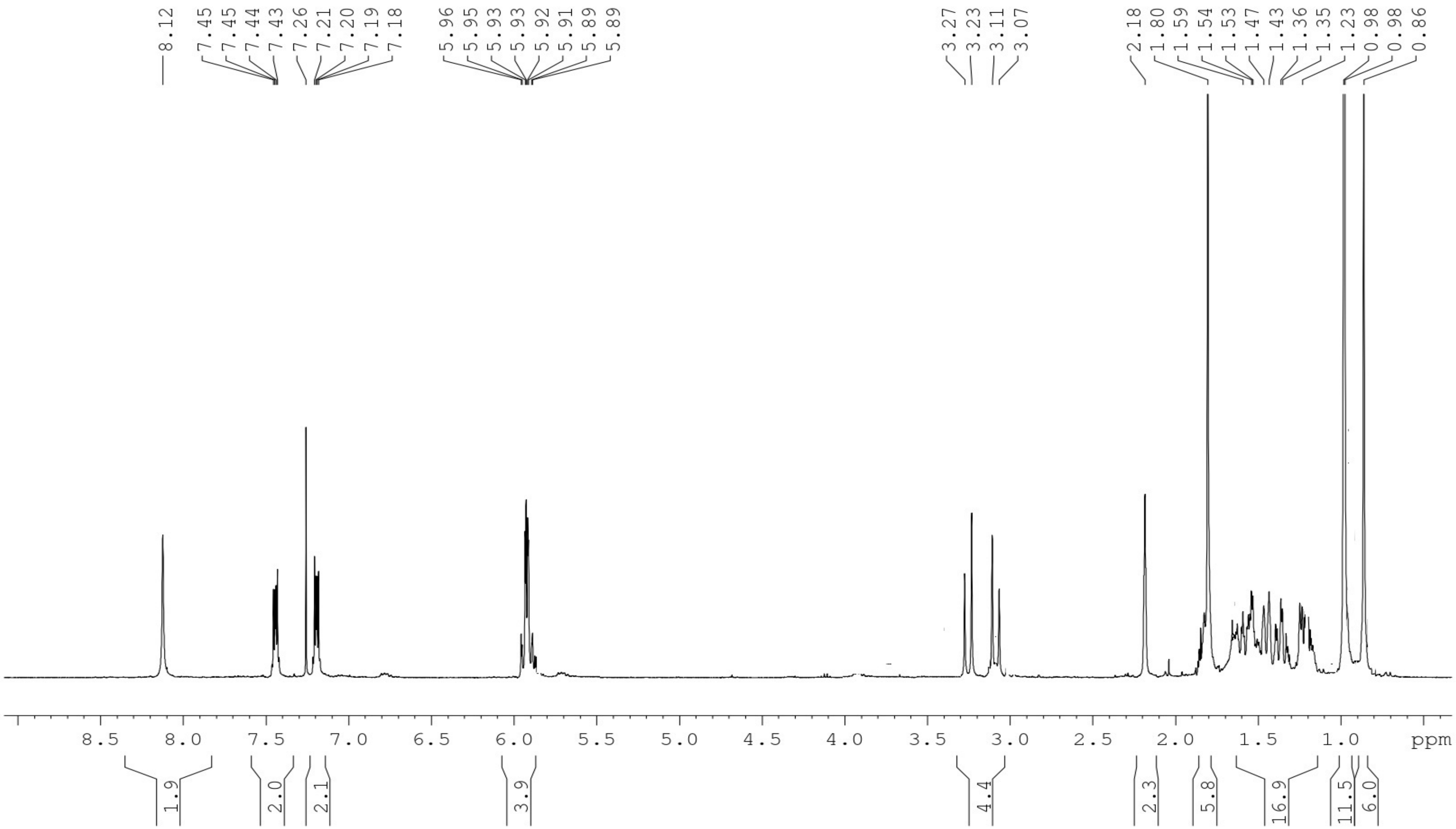

^{13}C NMR spectrum of compound 16

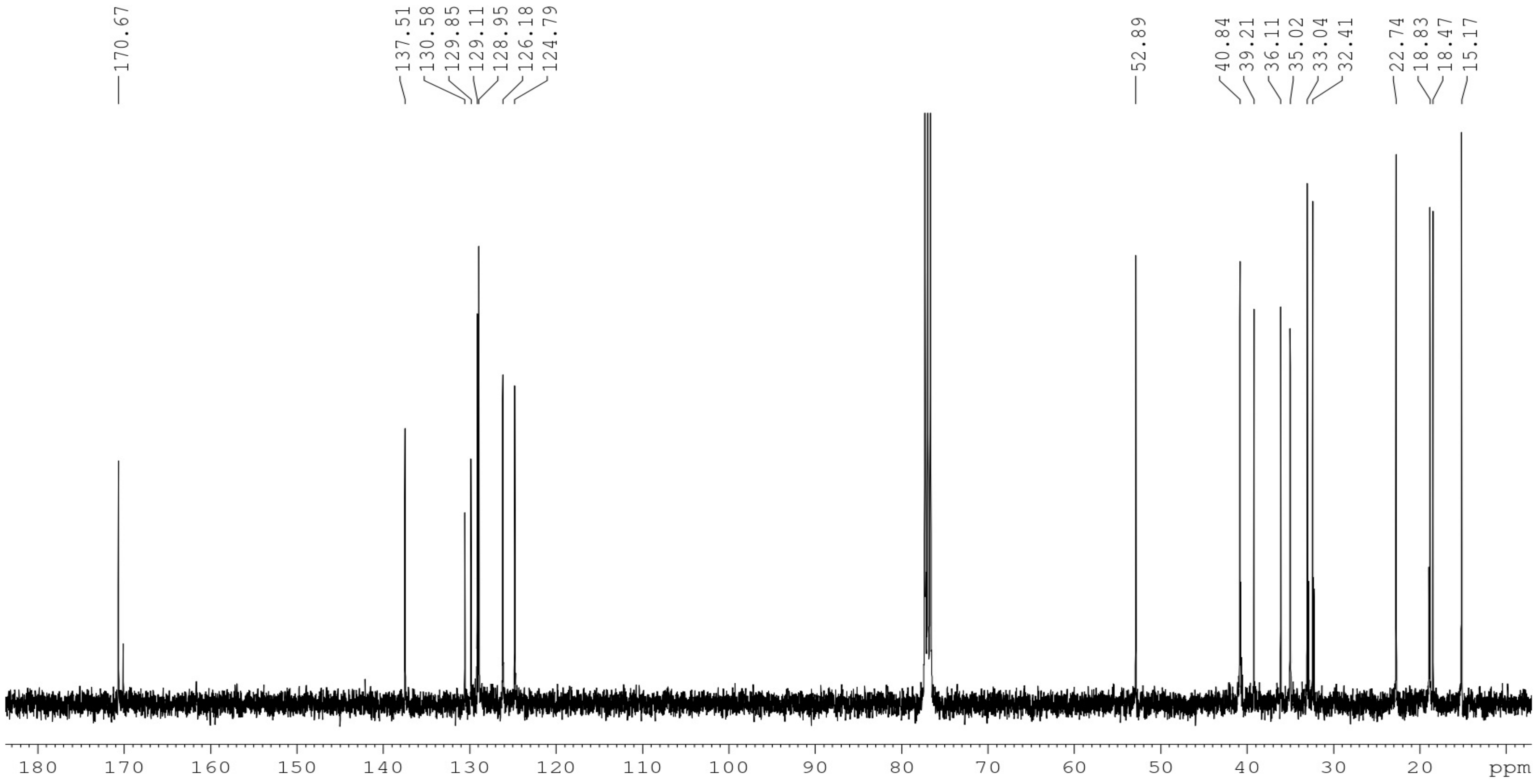

^{1}H NMR spectrum of compound 18

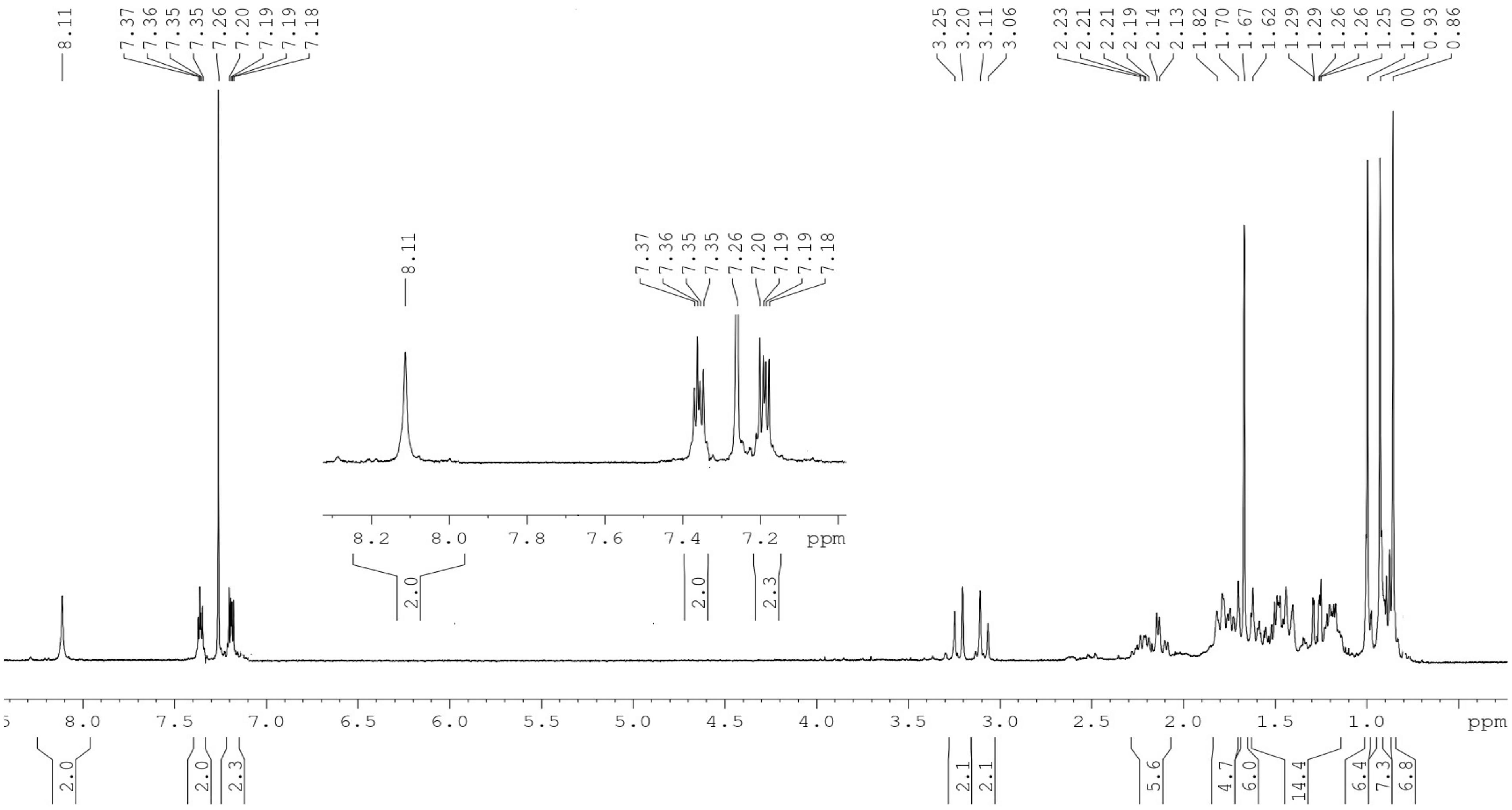

^{13}C NMR spectrum of compound 18

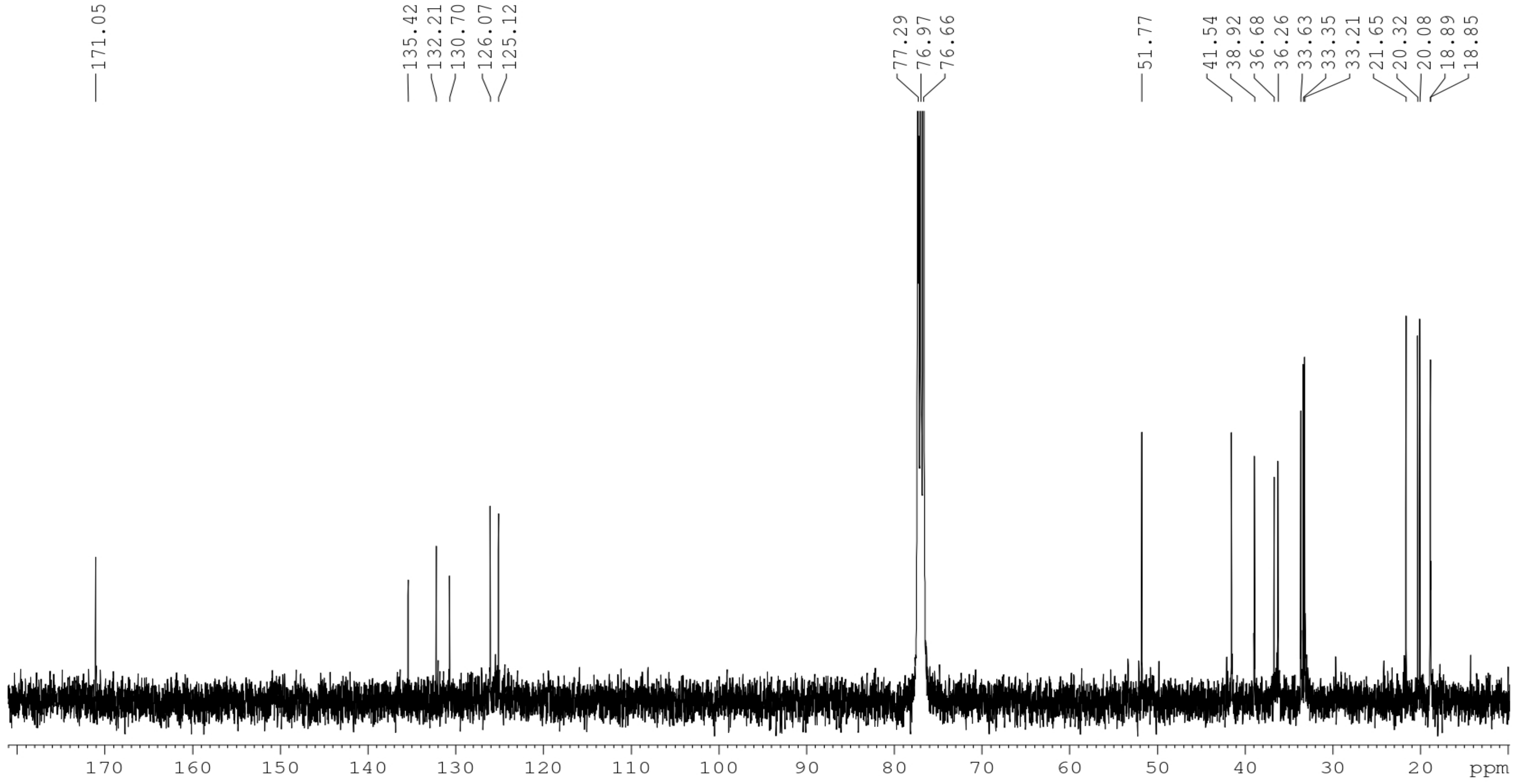

^{1}H NMR spectrum of compound 19

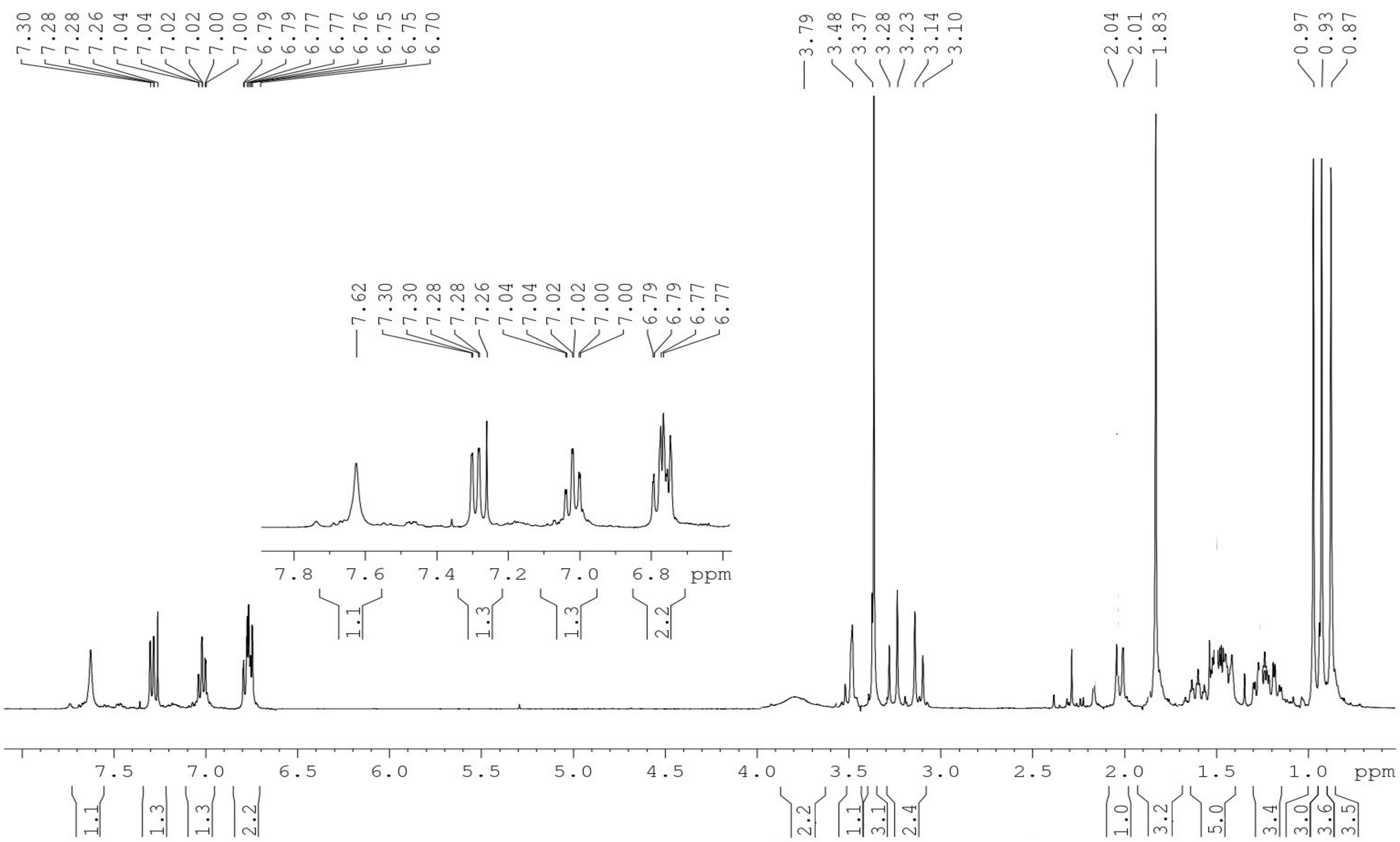

^{13}C NMR spectrum of compound 19

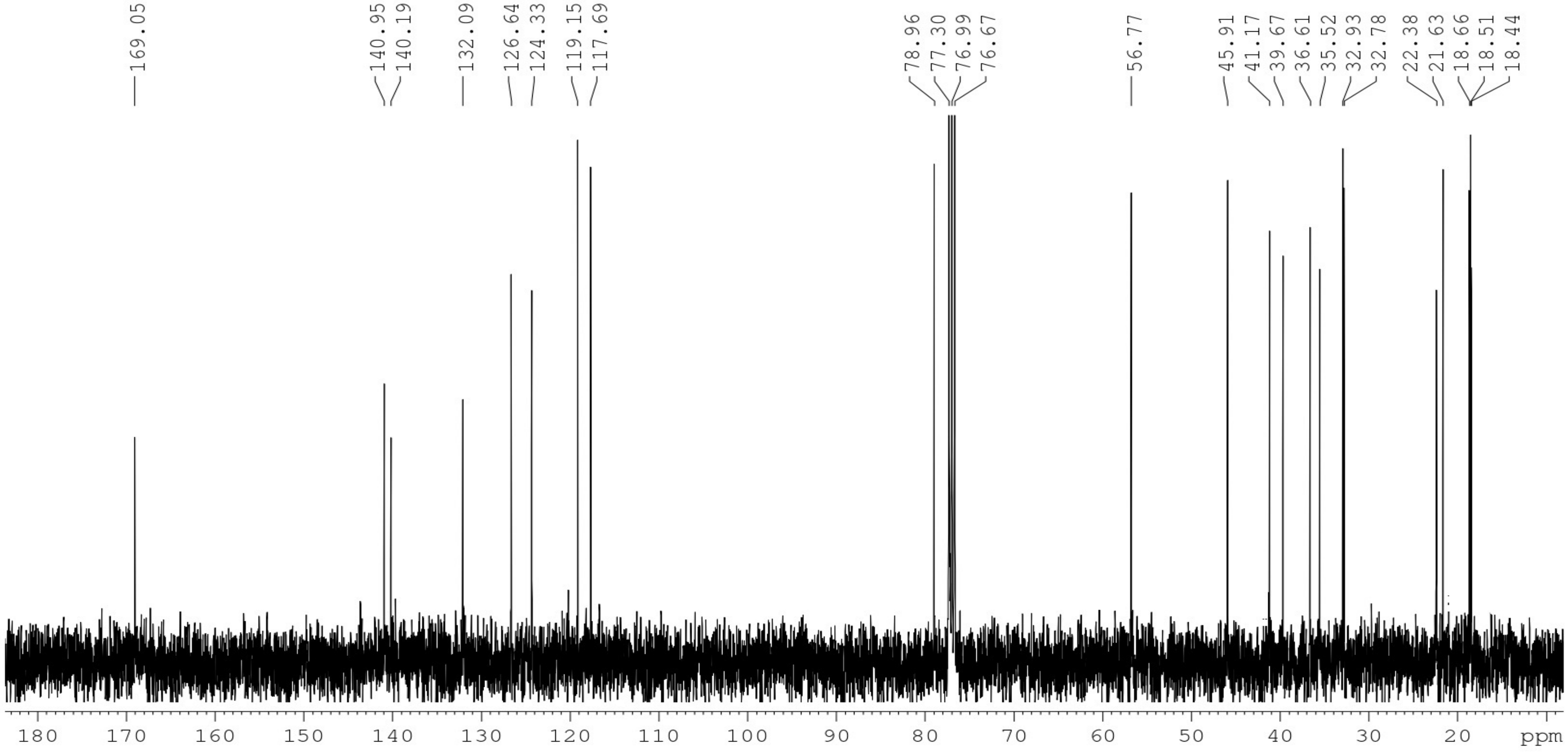

^{1}H NMR spectrum of compound 20

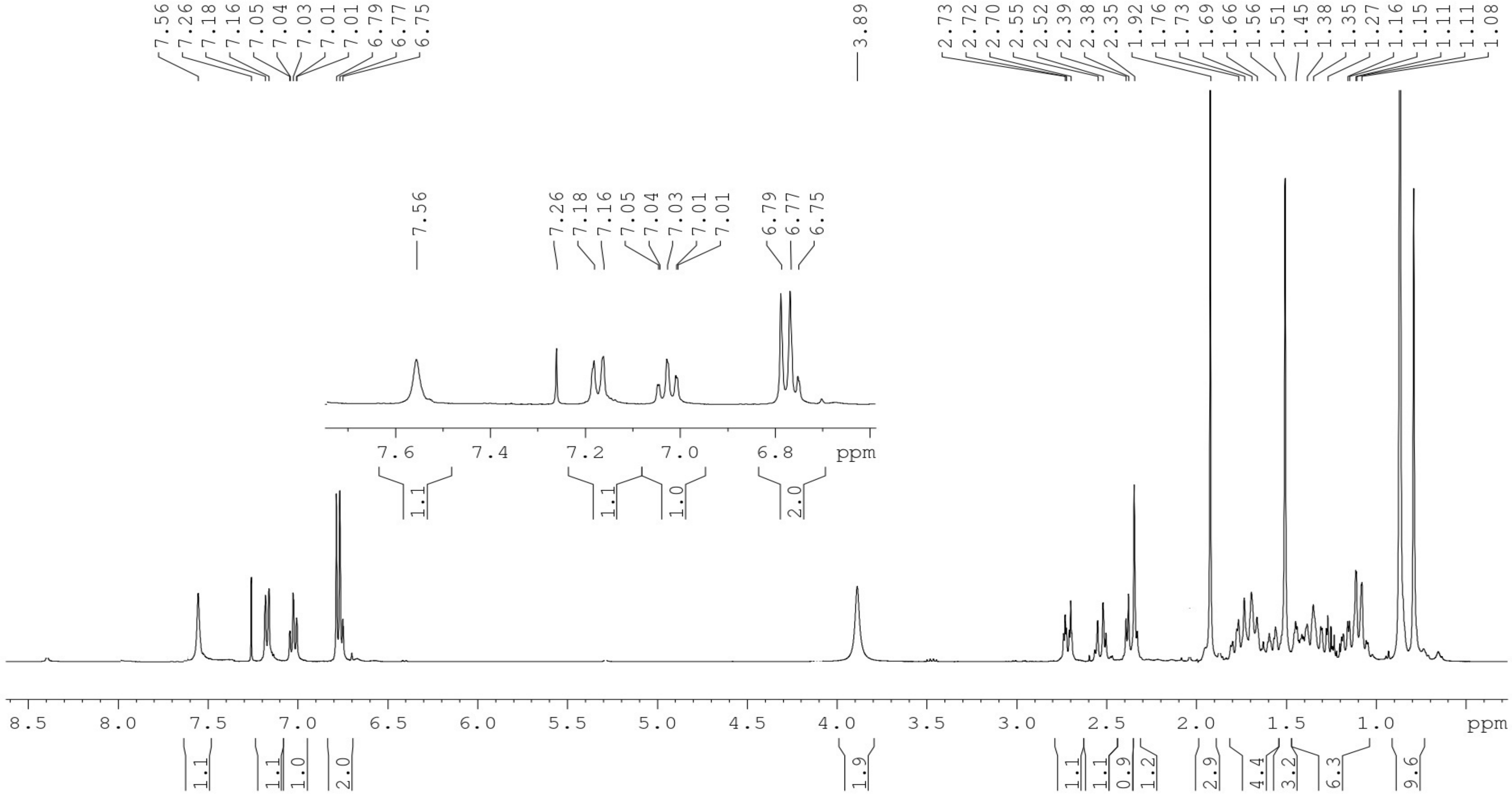

^{13}C NMR spectrum of compound 20

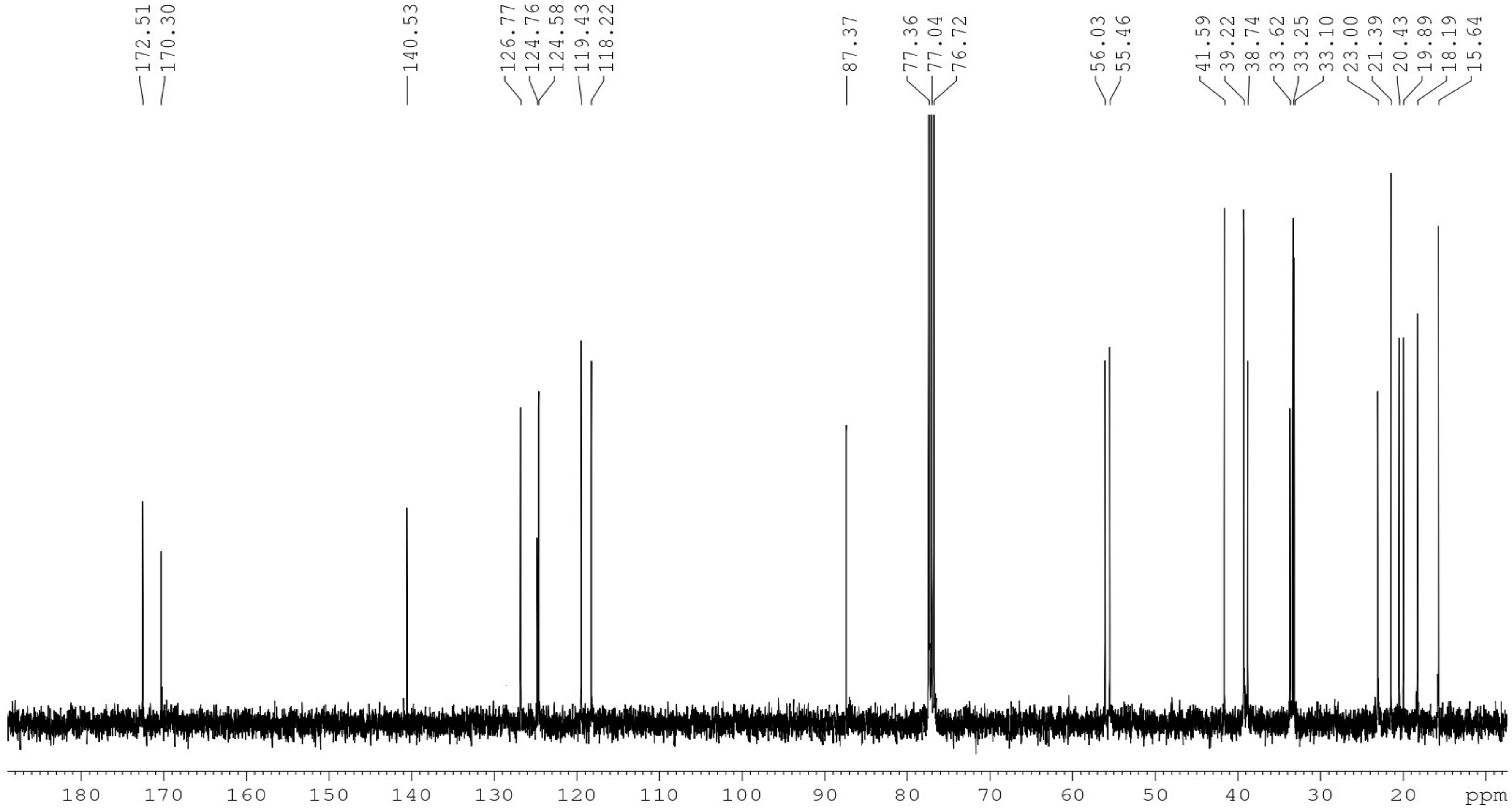

^{1}H NMR spectrum of compound 21

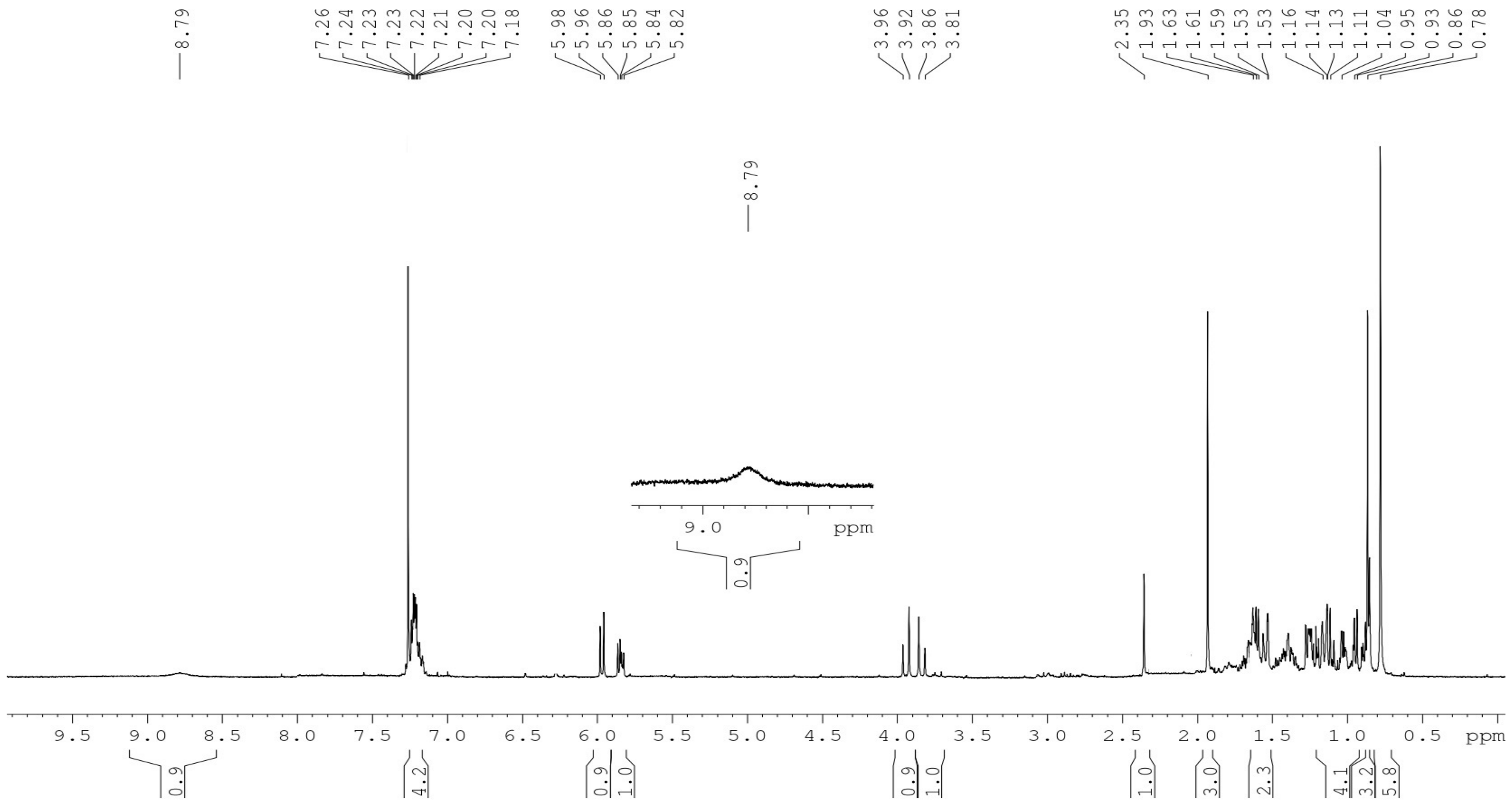

^{13}C NMR spectrum of compound 21

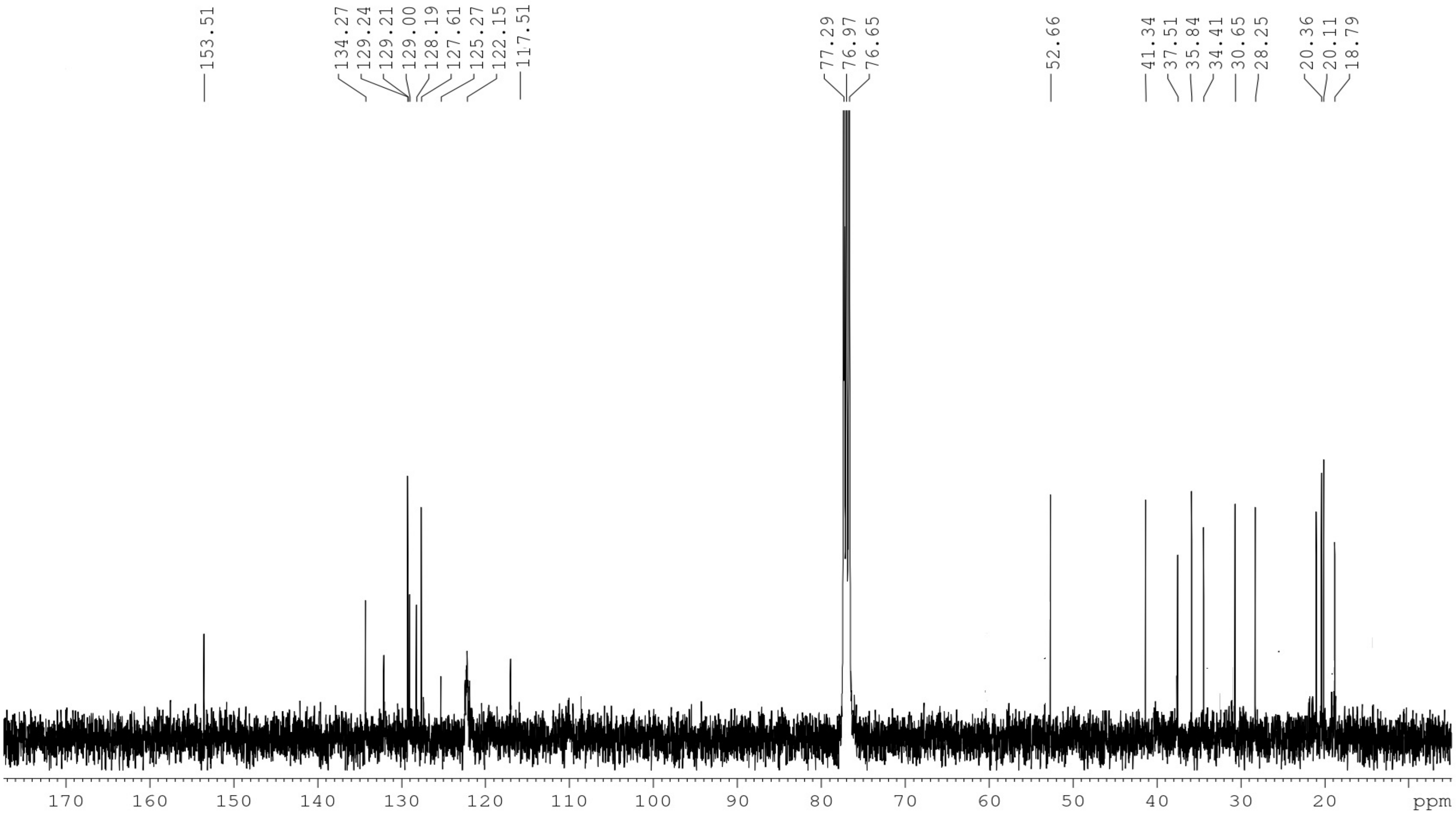

^{1}H NMR spectrum of compound 22

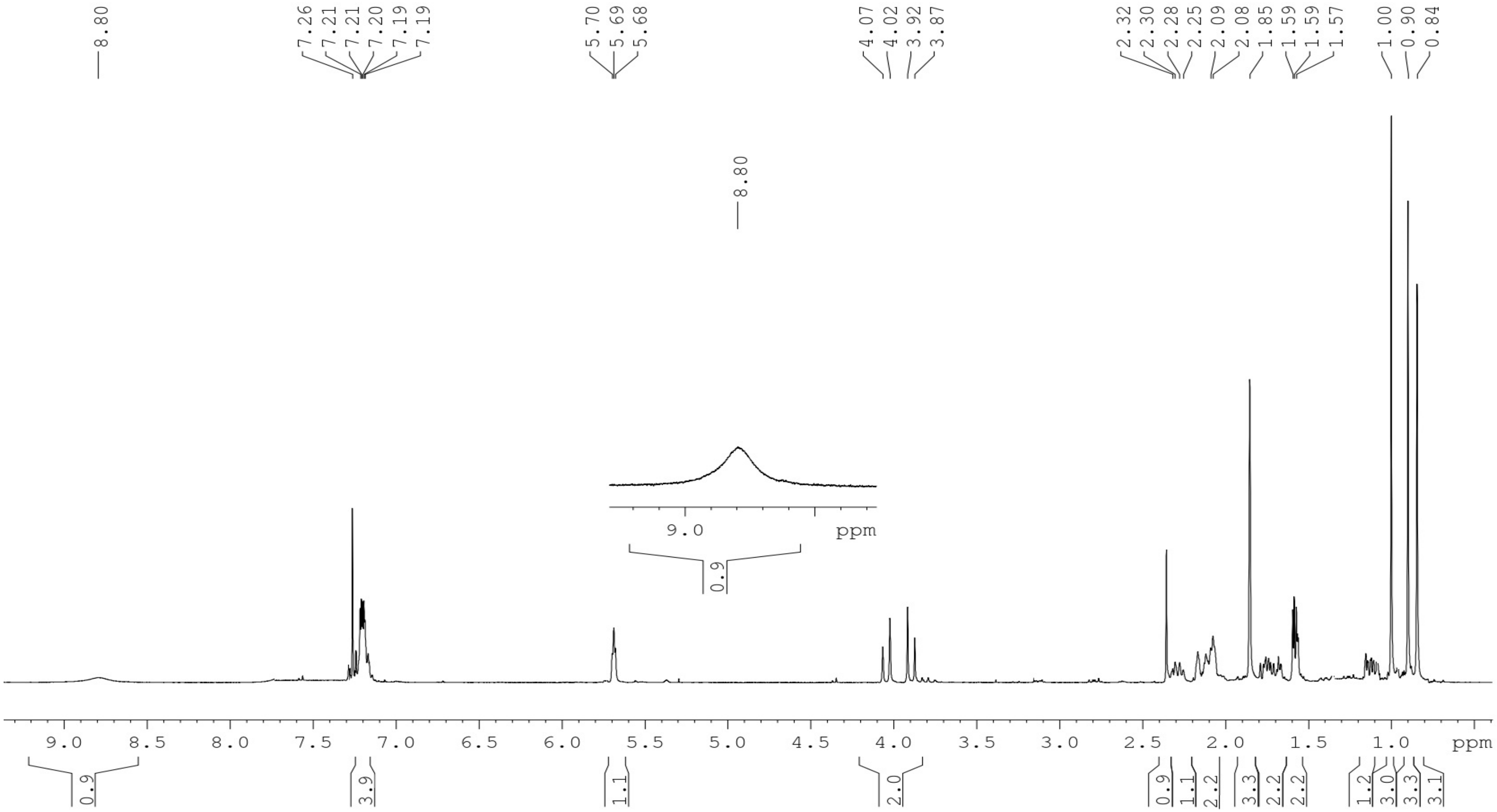

^{13}C NMR spectrum of compound 22

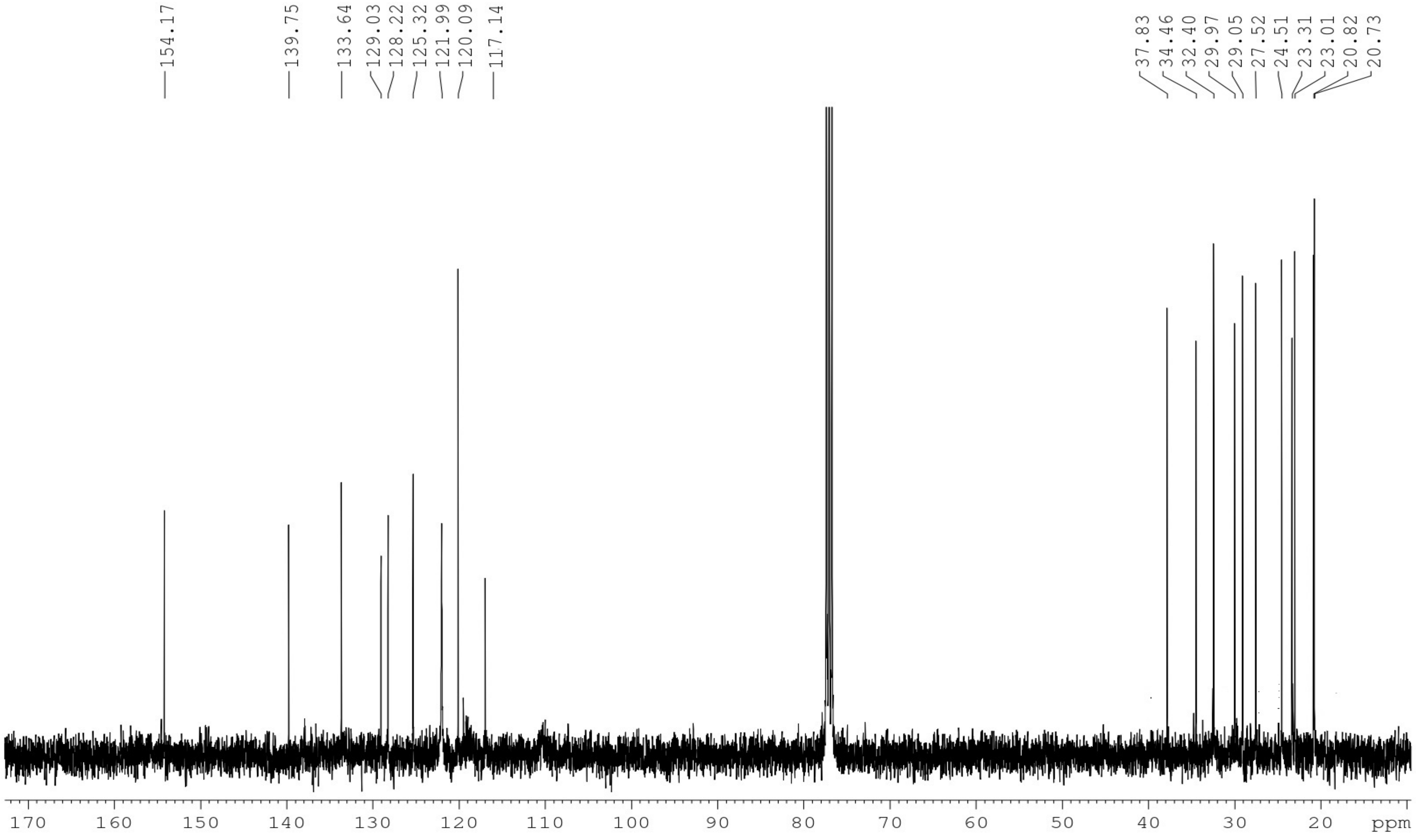

^{1}H NMR spectrum of compound 23

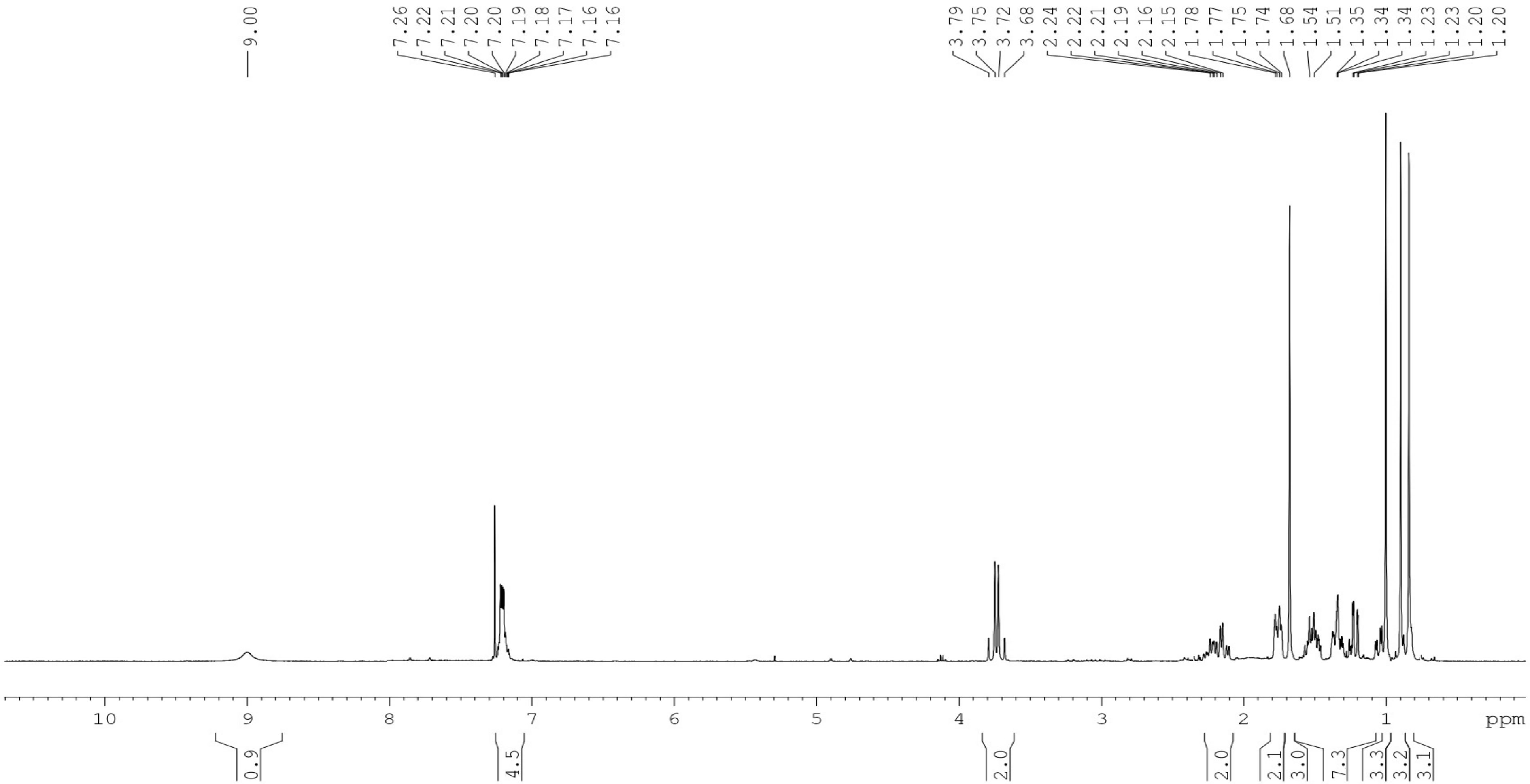

^{13}C NMR spectrum of compound 23

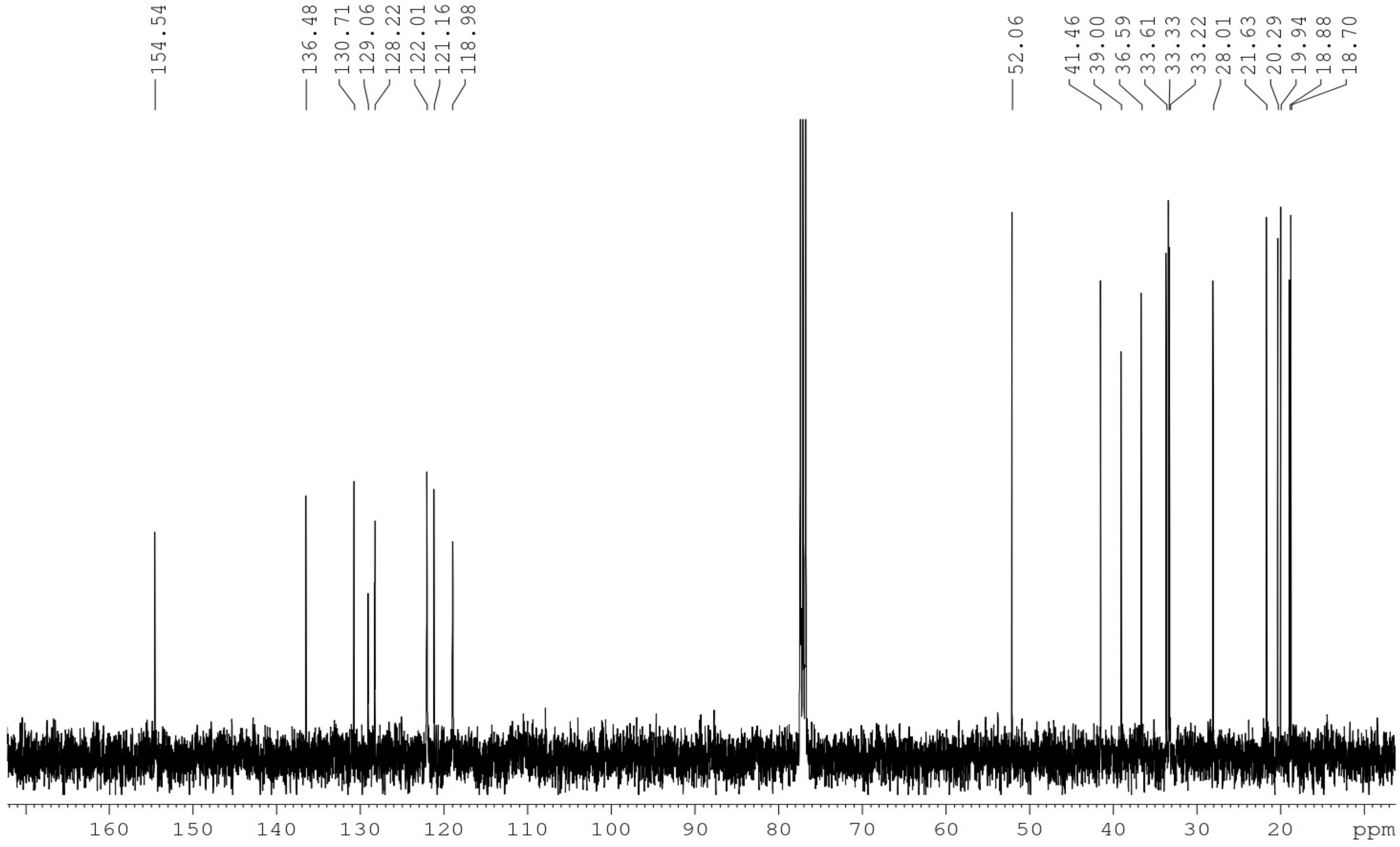

Supplement: Supplementary file 1 [file molecules-28-00933-s001.zip › molecules-2153553-supplementary.pdf]
